# Complete genome sequence of *Shigella* phage Moonfish isolated from Mid-Michigan

**DOI:** 10.1128/mra.01255-24

**Published:** 2025-05-20

**Authors:** Sundharraman Subramanian, Hazel McGuffin, Rachel Passage, John A. Dover, Kristin N. Parent

**Affiliations:** 1 Department of Biochemistry and Molecular Biology, Michigan State University 3078, East Lansing, Michigan, USA; 2 Department of Microbiology, Genetics & Immunology, Michigan State University 3078, East Lansing, Michigan, USA; 3 College of Science and the Environment, Lake Superior State University, Saulte Ste. Marie, Michigan, USA; Portland State University, Portland, Oregon, USA

**Keywords:** bacteriophage, genome, *Shigella*, podophage

## Abstract

A *Shigella* podophage, Moonfish, was isolated from a longhorn cattle ranch in Barry County, MI, USA. The 69,166-bp genome was annotated and places Moonfish in a similar category to N4-like phages. Host range studies show that Moonfish has a narrow host profile relative to similar phages.

## ANNOUNCEMENT

*Shigella flexneri* is the causative agent of shigellosis and is responsible for human disease worldwide (1). At the time we began our investigations, relatively few *Shigella* phages had been isolated compared with other enteric bacterial species. For example, there were only 34 known *Shigella* phages compared with thousands of *Escherichia coli* phages (2). Recently, we have been involved in *Shigella* phage-hunting activities to discover diverse species (3). *Shigella* podophages HRP29, Moo19, and B2 were isolated in Lincoln, NE, USA, as part of a high school phage-hunting exercise (4, 5). By contrast, no *Shigella* podophages have been isolated to date from our Michigan phage-hunting activities (6). As part of a graduate course in Fall 2018, we expanded our library of samples and found one podophage from Mid-Michigan.

**Fig 1:**
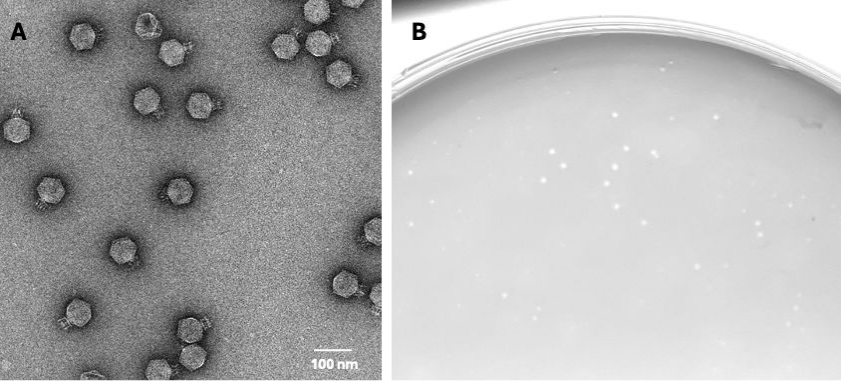
(A) Negatively stained micrograph of Moonfish, showing podophage morphology with short, non-contractile tails. Image was collected at MSU’s Cryo-EM Research Technology Support Facility on a Talos Arctica with a Ceta camera at 57,000× nominal magnification (pixel size 2.5 Å). (B) Plaque morphology of Moonfish.

Phage Moonfish was isolated from a water sample that stemmed from longhorn cattle trough water, collected on 4 September 2018 at GPS location: 42°26′50.5″N 85°14′22.0″W. The sample was collected in a sterile 50 mL conical tube and held at room temperature overnight. The sample was then filtered through a 0.22 µm syringe filter, and 500 µL of filtrate was propagated on *Shigella flexneri* serotype Y strain PE577 (7) using Miller’s LB Broth (Research Products International) in a top agar overlay at 37°C using standard protocols (3). Spot tests to gauge the host range were performed by combining bacterial cells in a double agar overlay method in triplicate, similar to the work previously described (6). Moonfish displayed a narrow host range with small, clear plaques (Table 1; Fig. 1) and is the only podophage we have isolated using serotype Y *Shigella,* with an unmodified O-antigen on the lipopolysaccharide (8); all others were isolated on and specific to strain CFS100, which is serotype 2a_2_ that has a highly decorated O-antigen (3, 5).

**TABLE 1:** Host range of Moonfish^*a*^

Bacterial host	Strain	Plaque (yes/no)	Strain reference
*Shigella flexneri*	PE577 (serotype Y)	Yes	(7)
	CFS100 (serotype 2a_2_)	No	(7)
*Salmonella typhimurium*	14028s	No	(9)
*Escherichia coli*	EV36 (K-12/K1 hybrid)	No	(10)
	MG1655 (K-12)	No	(11)
	REL606 (B strain)	No	(12, 13)
	C122 (C strain)	No	(14)

^*a*^ Non-pathogenic laboratory strains commonly used to characterize enteric phage isolates.

The Moonfish genome was extracted as described from particles concentrated from clarified lysate (6). Samples were prepared for whole-genome sequencing using the Illumina DNA Prep tagmentation kit and Illumina Unique Dual Indexes. Sequencing was performed on the Illumina NextSeq2000 platform using a 300 cycle flow cell kit to produce 2 × 150 bp paired reads. Read demultiplexing, read trimming, and run analytics were performed using DRAGEN version 4.2.7, an onboard analysis software on the NextSeq2000. The purified genomes were sequenced by SeqCoast Genomics (Portsmouth, NH, USA). In total, we had 347,539,022 input bases with an average input read length of 148 bp. We then trimmed reads using the trim sequences application within Galaxy (15) version 24.1 and assembled using SPAdes version 4.0.0 into one contig with a final genome coverage of 542.07 (15, 16). GeneMarkS (17) was used to identify open reading frames, and both sequences were manually annotated using BLAST (18), InterPro scan (19), and tRNAscan-SE (20) using default parameters.

Moonfish is a 69,166-bp podophage with a 43% GC content, 82 genes, 44 annotated genes, and 1 tRNA. Moonfish is most similar to the N4-like phages, with ~78% nucleotide identity to *Shigella* phage Moo19 (using BLAST alignments using standard databases, nr, etc.) (18). Negative stain electron microscopy using 1% uranyl acetate reveals that Moonfish has podophage morphology (Fig. 1) and a similar structure to that of phages Moo19 and B2 (21).

## Data Availability

The genome sequence and associated annotation for Moonfish were deposited under GenBank accession number PQ613263, and the raw reads are available through SRA under accession number PRJNA1219577.
